# Metabolic drivers of MASLD and MASH: from hormonal imbalance to fibrosis

**DOI:** 10.1007/s00125-026-06774-7

**Published:** 2026-06-20

**Authors:** Amalia Gastaldelli, Egeria Scoditti, Maria Paula Macedo

**Affiliations:** 1 Institute of Clinical Physiology, National Research Council, Pisa, Italy; 2 Centro Interdisciplinare Health Science, Scuola Superiore Sant’Anna, Pisa – UOC, Pisa, Italy; 3 UT Health San Antonio, Division of Diabetes, San Antonio, TX USA; 4 NOVA Medical School, Faculdade de Ciências Médicas (NMS, FCM), Universidade NOVA de Lisboa, Lisbon, Portugal; 5 APDP – Diabetes Portugal, Education and Research Center, Lisbon, Portugal; 6 iNOVA4Health, NOVA Medical School, Faculdade de Ciências Médicas (NMS, FCM), Universidade NOVA de Lisboa, Lisbon, Portugal

**Keywords:** Adipose tissue dysfunction, Carbohydrate metabolism, Glucagon, Incretin, Insulin, Insulin resistance, Insulin secretion in vivo, Insulin sensitivity, Lipid metabolism, MASLD, Metabolic dysfunction-associated steatotic liver disease, Metabolic inflammation, Metabolic physiology in vivo, Other hormones/action, Review

## Abstract

Metabolic dysfunction-associated steatotic liver disease (MASLD) and its more severe and aggressive form, metabolic dysfunction-associated steatohepatitis (MASH), represent liver-specific consequences of systemic metabolic dysfunction. This review synthesises current evidence on the metabolic and hormonal determinants of MASLD progression, with a particular focus on the mechanisms linking systemic dysfunction across multiple tissues to hepatic inflammation and fibrosis. MASLD pathogenesis is driven by complex interactions between insulin resistance, impaired glucose metabolism, dysfunctional adipose tissue lipolysis and altered hepatic de novo lipogenesis. Central to this process is the dysregulation of key metabolic hormones, including insulin and glucagon, whose imbalance disrupts both glucose and lipid fluxes, leading not only to hepatic steatosis but also to altered hepatic glucose handling, increased substrate delivery and reduced metabolic flexibility. This promotes lipid accumulation as well as glucotoxic and lipotoxic stress. These metabolic disturbances trigger hepatocellular injury and activate inflammatory signalling pathways, including adipokine-mediated crosstalk between adipose tissue and the liver, promoting hepatic stellate cell activation and ultimately leading to a state of chronic low-grade inflammation (metaflammation) that integrates hepatocyte stress, immune cell activation and hepatic stellate cell-driven fibrogenesis. This review also highlights sex-specific hormonal regulation and the gut–liver–adipose axis as determinants of disease heterogeneity and immunometabolic injury. By integrating mechanistic insights across glucose and lipid metabolism, tissues and hormonal pathways, this review underscores how systemic metabolic dysfunction is translated into progressive liver injury and fibrosis. Understanding these interconnected mechanisms of disease progression is essential for identifying therapeutic targets and advancing mechanism-based and precision medicine approaches for MASLD and MASH.

**Graphical Abstract:**

**Figure:**
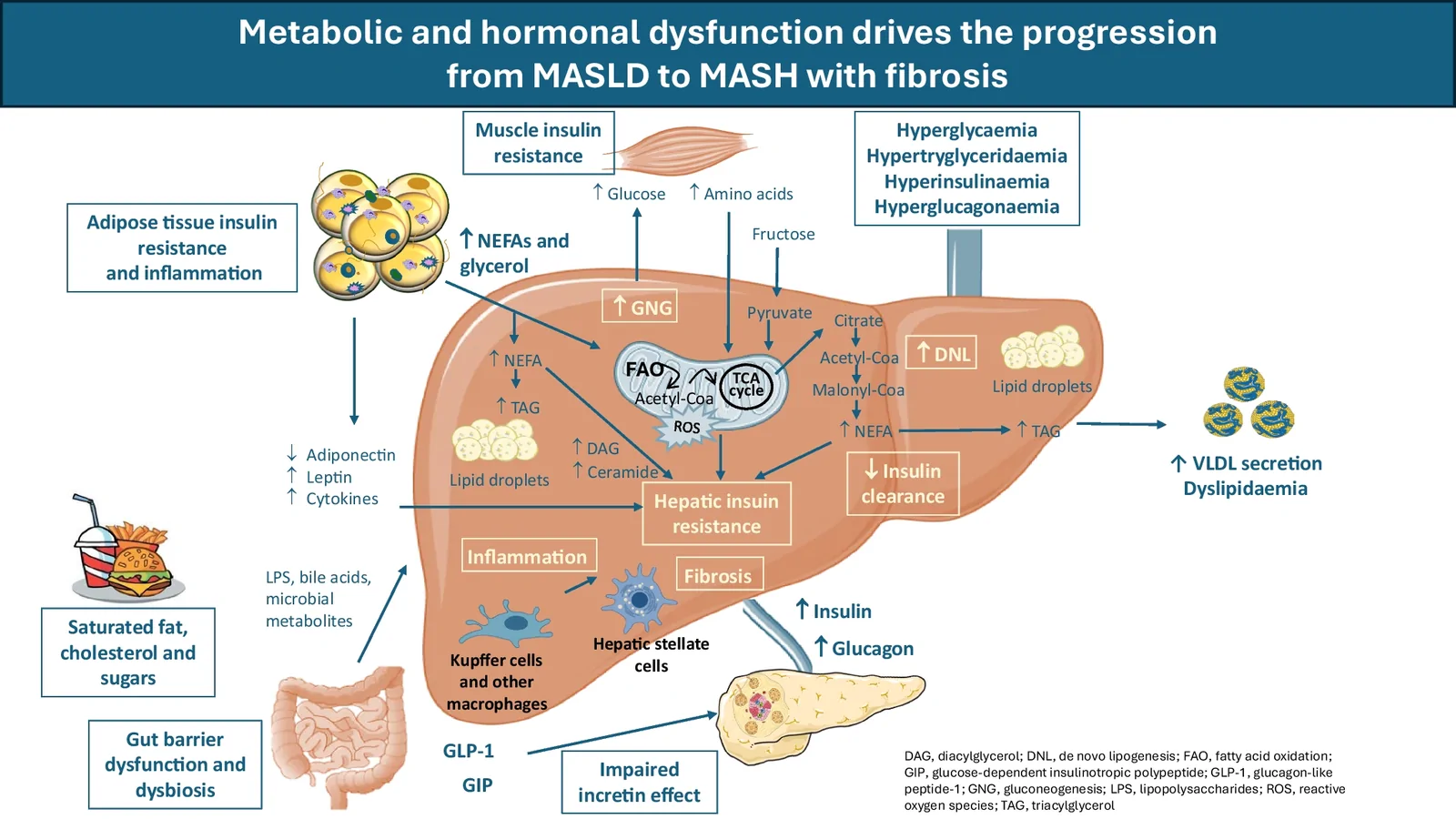

**Supplementary Information:**

The online version contains a slideset of the figures for download available at https://doi.org/10.1007/s00125-026-06774-7.

## Introduction

Metabolic dysfunction-associated steatotic liver disease (MASLD) is more than simple fat accumulation in the liver; it reflects alterations in metabolic, hormonal, inflammatory and fibrotic pathways [1]. The progression from isolated steatosis to metabolic dysfunction-associated steatohepatitis (MASH) and fibrosis is characterised by chronic low-grade inflammation, immune activation and maladaptive tissue repair. Insulin resistance, adipose dysfunction, altered hormone signalling and persistent inflammation converge to activate hepatic fibrogenic pathways. These processes vary among individuals [2], explaining disease heterogeneity: while many individuals remain stable, some progress to advanced fibrosis, cirrhosis or hepatocellular carcinoma, emphasising the need for mechanistic insights to guide risk stratification and targeted treatments.

The objective of this narrative review is to synthesise current evidence on the metabolic and hormonal drivers of MASLD and MASH, with a particular focus on the pathways linking systemic metabolic dysfunction to hepatic inflammation and fibrosis. Understanding these interconnected mechanisms is essential for advancing precision medicine approaches and improving clinical outcomes in context of this rapidly growing global health burden.

## Altered lipid and glucose metabolism in the development and progression of MASLD

### Adipose tissue dysfunction and hepatic metabolic dysregulation

Adipose tissue (AT) dysfunction is a central driver of both lipid overflow and systemic metabolic alterations in MASLD, contributing to hepatic steatosis and injury. By coordinating NEFA release, uptake and storage as triacylglycerols, AT adapts to changes in energy balance across feeding–fasting cycles, thereby contributing to metabolic homeostasis.

Subcutaneous adipose tissue (SAT) plays a key protective role by buffering excess energy and limiting ectopic lipid deposition in the liver [3]. This buffering capacity underlies metabolic flexibility, that is, the ability of tissues to adjust substrate use in response to nutrient availability and hormonal signals, switching efficiently between glucose and lipid oxidation [4]. Such regulation depends on coordinated hormonal control, including insulin-mediated suppression of lipolysis and catecholamine-driven lipid mobilisation [5, 6].

**Fig. 1:**
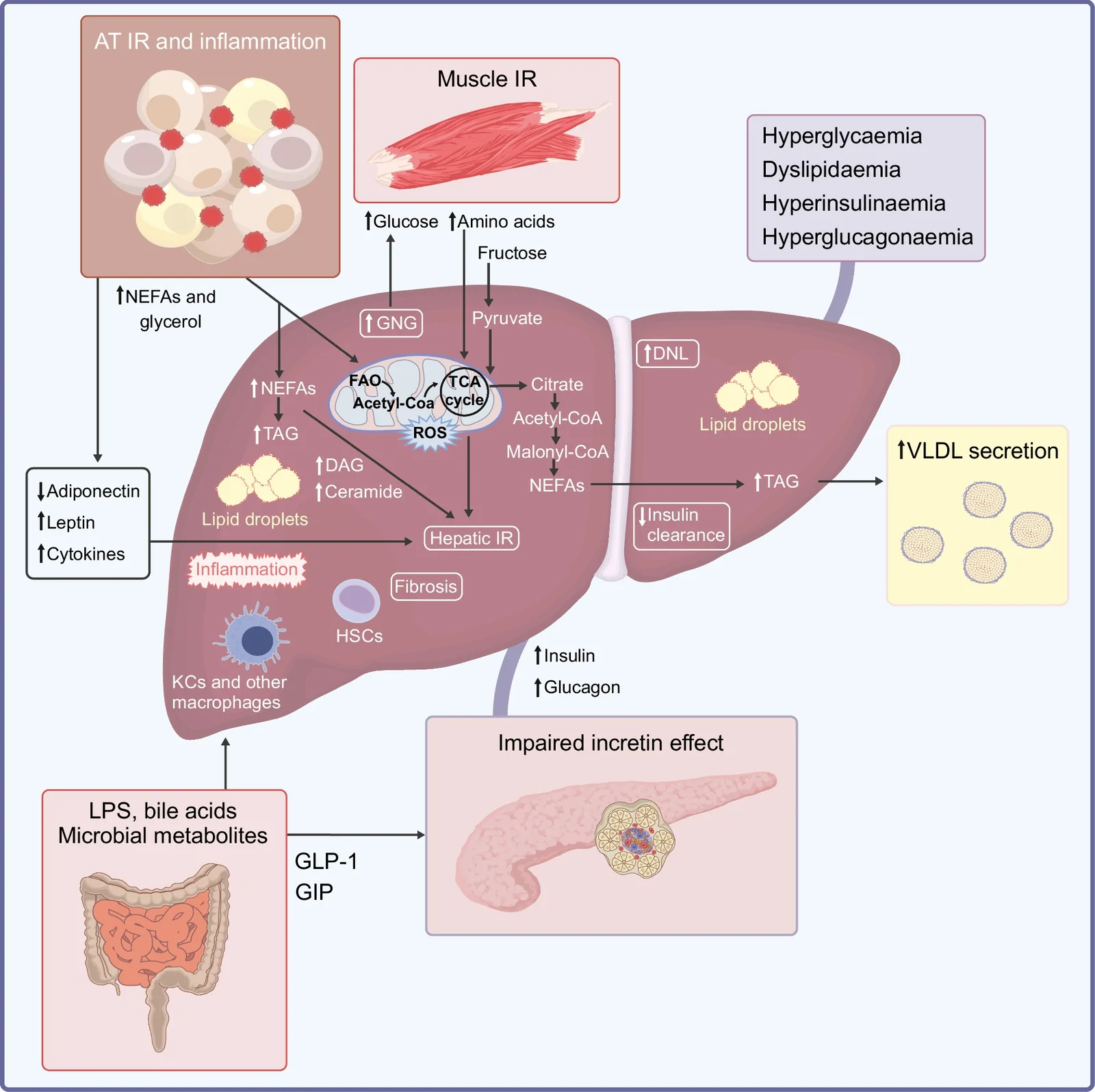
Metabolic network linking substrate (NEFAs, glycerol, amino acids, fructose) excess, peripheral and liver insulin resistance, inflammation and fibrosis in MASLD/MASH. MASLD development and progression to MASH are driven by the accumulation of triacylglycerol (TAG) and toxic metabolic intermediates resulting from excess substrate influx, de novo lipogenesis (DNL) and metabolic stress, which leads to insulin resistance (IR) and steatosis. Systemic inputs of substrates to the liver include obesity-driven insulin-resistant AT, which increases the hepatic delivery of NEFAs and glycerol owing to unrestrained lipolysis. Excess hepatic NEFAs promote esterification into TAG for storage, secretion as VLDL to reduce lipotoxicity, or oxidation in the mitochondria. Muscle insulin resistance causes impaired glucose uptake and promotes protein catabolism, contributing to nutrient input to the liver. Excess substrate and energy availability from FAO, carbohydrates (e.g. fructose, a staple of Western diets) and amino acids stimulates the generation of NEFAs through DNL, which is a major contributor to liver steatosis. Mitochondrial overload generates incomplete oxidative byproducts including reactive oxygen species (ROS). Excessive hepatic NEFA influx, when not balanced by FAO, drives the accumulation of toxic lipid intermediates such as diacylglycerol (DAG) and/or ceramide [8, 38, 138]. Hepatic lipid accumulation and lipotoxicity trigger insulin resistance, which is accompanied by impaired insulin clearance. Under conditions of hepatic insulin resistance and increased substrate availability, gluconeogenesis (GNG) is also stimulated, contributing to high HGP and hyperglycaemia. Hyperinsulinaemia develops as a result of adaptive beta cell responses to peripheral insulin resistance and to reduced hepatic insulin clearance, and further promotes DNL. In insulin-resistant states, glucagon release from the pancreas is unsuppressed, resulting in high concentrations of glucagon reaching the liver. Moreover, the gut-derived incretin hormones GLP-1 and GIP are impaired in MASLD. Hepatocyte stress and metabolic imbalance are further driven by an aberrant pattern of adipokines and gut-derived signals, including bile acids, lipopolysaccharides (LPS) and microbial metabolites. These processes activate proinflammatory and profibrogenic signalling pathways—including NF-κB, NLRP3 and TLR—in hepatocytes, resident macrophages (Kupffer cells [KCs]) and monocyte-derived macrophages, resulting in increased production of cytokines, chemokines and growth factors. Under these stimuli, hepatic stellate cells (HSC) are activated to produce a fibrogenic extracellular matrix. This figure is available as part of a downloadable slideset

With chronic energy excess, SAT storage capacity becomes saturated, leading to impaired lipid sequestration and increased NEFA release [3, 7]. These excess NEFAs are taken up by non-adipose tissues, promoting lipotoxicity, inflammation and systemic insulin resistance [7–10] (Fig. 1). Stable isotope tracer and positron emission tomography (PET) studies indicate that increased AT-derived NEFA flux is a major contributor to hepatic lipid accumulation, with systemic NEFAs contributing ~60% of intrahepatic triacylglycerols [11–14].

Visceral adipose tissue (VAT) is more strongly associated with insulin resistance and hepatic metabolic dysfunction than SAT, even in lean individuals with MASH [10, 12, 15]. VAT exhibits higher intrinsic lipolytic activity, driven by increased catecholamine sensitivity [6] and reduced insulin-mediated suppression [16], resulting in greater NEFA release. Accordingly, noradrenaline (norepinephrine)-stimulated NEFA levels are higher in VAT than SAT [6] and are further increased in individuals with MASLD [17]. Despite contributing less to total systemic NEFA flux than SAT, VAT may exert a disproportionate hepatic effect owing to its direct drainage into the portal circulation, exposing the liver to higher local concentrations of NEFAs and proinflammatory mediators [15, 18].

Consistent associations between VAT and hepatic steatosis support a key role for VAT in disease pathogenesis, although causality independent of whole-body metabolic dysfunction remains unproven [10, 12, 15, 17]. In contrast, SAT shows weaker or less consistent associations than VAT, indicating depot-specific differences in the contribution of AT to hepatic lipid accumulation [6].

### Alterations in glucose metabolism during MASLD progression

MASLD is associated not only with altered lipid metabolism and lipotoxicity, but also with disturbances in glucose metabolism. The presence of MASLD increases the risk of type 2 diabetes [19] and, conversely, type 2 diabetes and impaired glucose tolerance are linked to more severe liver disease [20]. These associations support a close interplay between glucose metabolism and disease progression, although the extent to which this relationship reflects direct causal mechanisms, as opposed to shared underlying metabolic disturbances such as insulin resistance, remains to be fully established.

Most individuals with MASLD display fasting hyperglycaemia [12, 21, 22], which primarily reflects excessive hepatic glucose production (HGP) owing to hepatic insulin resistance. In contrast, postprandial hyperglycaemia results from a combination of hepatic/peripheral insulin resistance and an inadequate beta cell secretory response, with beta cell function already beginning to decline in impaired glucose tolerance [23, 24]. In MASH, elevated fasting HGP is largely driven by increased gluconeogenesis, fuelled by excess delivery of substrates such as lactate, glycerol and amino acids from insulin-resistant tissues [12, 25–27] (Fig. 1). The magnitude of the gluconeogenic flux is not associated with the degree of steatosis but rather with fibrosis stage [26], indicating that reduced hepatic metabolic flexibility, rather than steatosis per se, drives hyperglycaemia and MASLD progression. This establishes a feed-forward loop in which hyperglycaemia and insulin resistance further exacerbate liver disease progression.

### Mitochondrial dysfunction in liver and AT

The initial response of liver mitochondria to excess lipids and sugars appears to be adaptive. In vivo tracer studies and ex vivo respirometry of liver tissues have shown either no change or increased hepatic fatty acid oxidation (FAO) in individuals with MASLD compared with control individuals [22, 27–31], supporting a compensatory response to lipid overload, although this is accompanied by mitochondrial uncoupling [30]. In parallel, glucotoxicity may further exacerbate mitochondrial stress by increasing tricarboxylic acid (TCA) cycle flux and promoting redox imbalance through excess reducing equivalents. However, with progression to MASH, this adaptive capacity is lost, resulting in a decline in mitochondrial FAO, higher mitochondrial mass, lower maximal respiration, elevated oxidative stress and compromised mitochondrial turnover [30, 32]. This is accompanied by disruption of TCA cycle homeostasis, contributing to reduced metabolic flexibility and bioenergetic failure (Fig. 1).

In obesity, adipocyte mitochondrial dysfunction, characterised by alterations in morphology and dynamics (fusion/fission) and impaired oxidative phosphorylation and ATP production, further reduces bioenergetic capacity, limits NEFA use and sustains oxidative stress [33–36]. Conversely, restoration of AT mitochondrial function enhances FAO and attenuates hepatic steatosis [35].

Together, adipose-derived lipid overflow, impaired glucose homeostasis and mitochondrial dysfunction establish a systemic metabolic milieu that promotes hepatic vulnerability to inflammatory and immune-mediated injury.

## From metabolic dysfunction to inflammation and fibrosis in MASLD

### Metabolic stress and hepatocellular injury as the initiating event

The convergence of excess lipid and glucose fluxes promotes hepatocellular inflammatory signalling owing to lipotoxicity and glucotoxicity. Chronic exposure to NEFAs and lipotoxic metabolites, such as diacylglycerol (DAG) and ceramide, activates stress-responsive pathways in hepatocytes, including c-Jun N-terminal kinase (JNK) and NF-κB signalling, leading to cytokine/chemokine production and immune cell recruitment [37]. Accumulation of DAGs within hepatocytes activates novel protein kinase C (PKC) isoforms, particularly PKCε, which interfere with insulin receptor signalling and downstream Akt activation [38]. Ceramides further exacerbate insulin resistance by inhibiting Akt phosphorylation and promoting mitochondrial dysfunction and apoptosis [2, 39].

These inflammatory signals promote activation of hepatic stellate cells (HSCs), the principal fibrogenic cells in the liver, as well as extracellular matrix deposition and the progression of fibrosis [40]. Activated HSCs transdifferentiate into myofibroblast-like cells, secrete extracellular matrix proteins including collagen, and drive architectural remodelling, ultimately contributing to fibrosis [41].

Crosstalk between macrophages, hepatocytes and HSCs links metabolic stress to fibrogenesis. Fibrosis is now recognised as a dynamic process and reflects a metabolically driven wound-healing response rather than a linear inflammatory cascade. It is driven by repetitive cycles of injury and repair, where insulin resistance, AT inflammation and lipotoxicity sustain hepatocyte damage and macrophage activation, promoting HSC-mediated fibrogenesis [42]. Importantly, fibrosis stage, rather than inflammation, is the main predictor of clinical outcomes [43]; early stages may stabilise or regress with improvement in metabolic dysfunction, underscoring the dynamic and potentially reversible nature of fibrogenesis [42].

### Adipose tissue-driven inflammation as an upstream trigger of hepatic injury

**Fig. 2:**
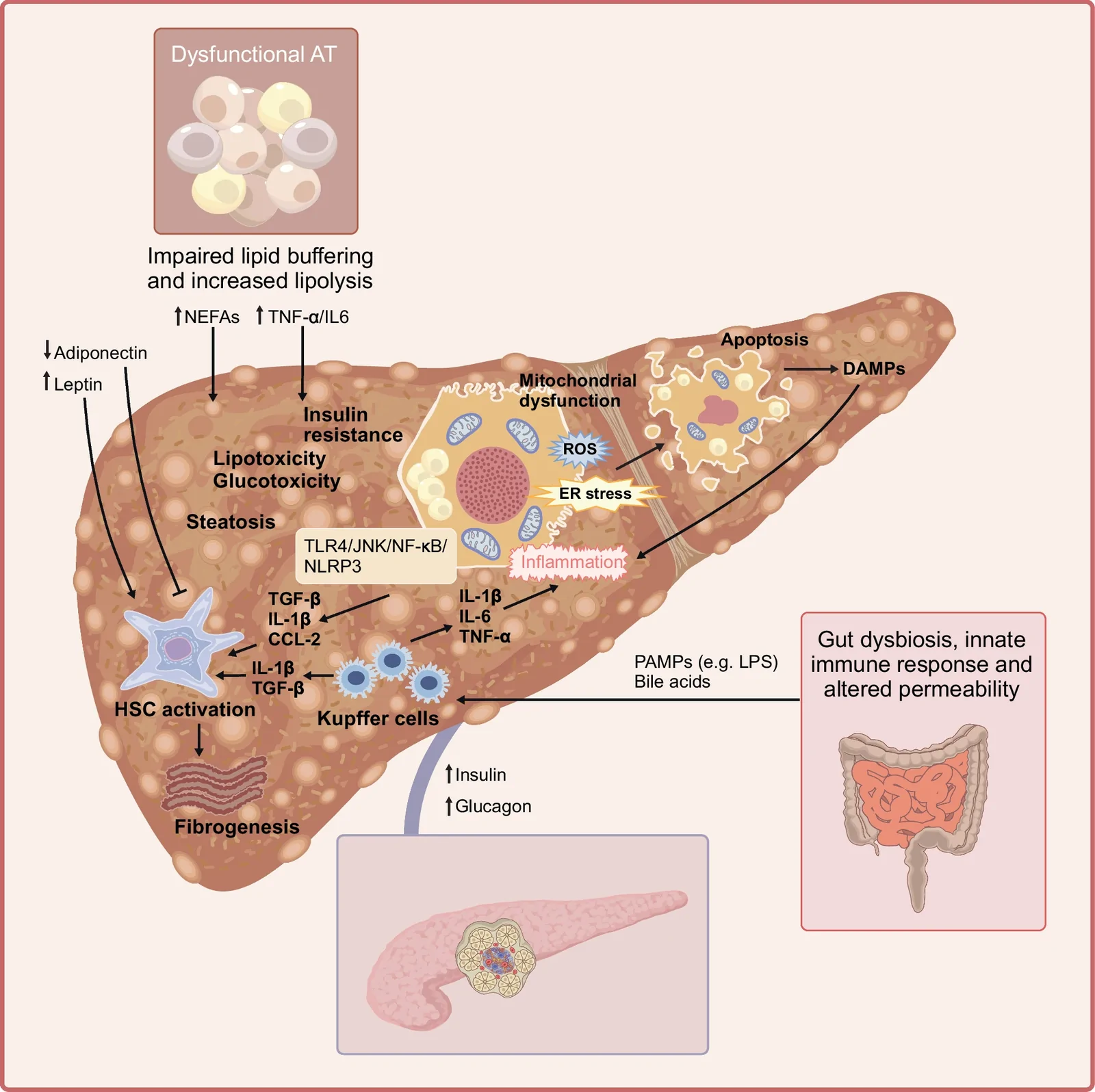
Mechanisms of hepatic fibrogenesis. Pathophysiological mechanisms of hepatic fibrosis are driven by dysfunctional AT and include increased lipotoxicity, ectopic fat deposition, enhanced release of proinflammatory cytokines (e.g. TNF-α, IL-6, leptin) and reduced levels of anti-inflammatory adipokines such as adiponectin. In conjunction with deregulated insulin and glucagon action, the resulting lipotoxicity and glucotoxicity induce endoplasmic reticulum (ER) stress, mitochondrial dysfunction and chronic inflammation in the liver. This environment promotes activation of HSCs and Kupffer cells, leading to extracellular matrix deposition and progression of fibrosis. Additional contributions arise from the gut, where dysbiosis and altered intestinal permeability promote the translocation of lipopolysaccharides (LPS) and other pathogen-associated molecular patterns (PAMPs) to the liver, along with alterations in bile acids, further amplifying inflammation and fibrogenic pathways. DAMPs, damage-associated molecular patterns; ROS, reactive oxygen species. This figure is available as part of a downloadable slideset

AT tissue dysfunction is a key driver of lipid overflow and promotes systemic inflammation (Fig. 2). In obesity and insulin resistance, AT undergoes immune cell infiltration and releases proinflammatory adipokines, including TNF-α, IL-6 and leptin, while secreting reduced levels of anti-inflammatory mediators such as adiponectin [3, 6, 44].

Importantly, adipose tissue-derived inflammatory mediators act as an upstream sensitising signal that lowers the threshold for hepatic immune activation, independently of lipid accumulation per se [3]. This establishes a mechanistic link between systemic metabolic dysfunction and intrahepatic inflammation beyond substrate overload alone.

### Hepatic innate immunity and macrophage activation

Innate immune activation is central to disease pathogenesis. Hepatic immune responses integrate metabolic stress signals, driving inflammation and fibrogenesis. MASLD is characterised by chronic low-grade inflammation, or ‘metaflammation’, driven by nutrient excess, insulin resistance and lipotoxic stress. This process links hepatocyte injury with innate immune activation and HSC fibrogenesis, promoting progression from steatosis to fibrosis [2, 42] (Fig. 2).

Kupffer cells, the liver-resident macrophages, sense lipid overload, cholesterol crystals and damage-associated molecular patterns (DAMPs), triggering Toll-like receptor 4 (TLR4)–NF-κB and NLRP3 inflammasome pathways. Activated Kupffer cells release cytokines (TNF-α, IL-1β) and chemokines (CCL-2), driving recruitment of monocyte-derived macrophages, expanding the hepatic macrophage pool and promoting a pro-fibrogenic phenotype [45] (Fig. 2).

Triggering receptor expressed on myeloid cells 2 (TREM2), a lipid-sensing receptor on Kupffer cells and recruited macrophages, regulates lipid handling, efferocytosis and inflammation resolution by promoting a reparative, lipid-associated macrophage phenotype. TREM2^+^ macrophages accumulate in crown-like structures around injured hepatocytes and lipid remnants, where they contribute to lipid clearance, modulation of inflammatory signalling and tissue remodelling, thereby influencing fibrosis progression. In addition, TREM2⁺ macrophages facilitate crosstalk with endothelial and stromal cells, supporting coordinated responses during liver injury and repair [46, 47].

### The gut–liver–adipose axis: amplifying metabolic inflammation and fibrosis

Beyond adipose- and liver-intrinsic mechanisms, the gut–liver axis is a key modulator of hepatic inflammation and fibrogenesis in MASLD [37, 48]. Increased intestinal permeability and dysbiosis promote translocation of pathogen-associated molecular patterns (PAMPs), including lipopolysaccharides, which activate Kupffer cells through TLR signalling, thereby amplifying hepatic metaflammation [48]. In preclinical models, obesity- and diabetes-associated gut dysbiosis induce aberrant innate immune activation, including neutrophil hyperactivation, contributing to barrier dysfunction and endotoxaemia; inhibition of neutrophil activity restores barrier integrity and attenuates liver and adipose inflammation and steatosis [49].

Microbiota-driven alterations in bile acid composition are associated with fibrosis stage in individuals with MASLD and MASH [50] and influence farnesoid X receptor (FXR)- and Takeda G protein-coupled receptor 5 (TGR5)-dependent signalling, affecting hepatic lipid metabolism, immune activation and HSC behaviour, as demonstrated in mouse models of liver fibrosis [51]. Gut-derived metabolites interact with liver and AT, impacting insulin signalling, metabolism and inflammation [52, 53] and reinforcing disease progression. Gut-derived metabolites and hormones, including incretins, further integrate liver–adipose crosstalk and immunometabolic responses. However, human evidence on the role of gut dysbiosis in MASLD remains largely associative, and causality requires validation in interventional studies.

## Hormonal dysregulation in MASLD and MASH

MASLD is characterised by hormonal dysregulation, which drives hepatic inflammation and fibrogenesis. Disturbances in insulin and glucagon signalling represent key mechanistic links between systemic metabolic dysfunction and liver disease progression. Disruption of this coordinated axis amplifies hepatocellular stress, immune activation and fibrogenesis (Fig. 2).

### Tissue-specific insulin resistance in MASLD

MASLD is characterised by insulin resistance involving multiple organs and tissues, such as liver, skeletal muscle and AT, with each contributing distinct yet interconnected defects [21, 22, 29, 54, 55]. In the liver, defective insulin action fails to suppress HGP, promoting hepatic lipid accumulation and lipotoxicity via increased adipose-derived NEFA flux [5, 21, 26, 29, 38]. In skeletal muscle, insulin resistance reduces postprandial glucose uptake [12, 21, 22, 29]. These defects worsen with the progression of fibrosis [26, 56, 57] and vary across individuals and throughout the day [5, 12, 21, 22, 29, 58], contributing to marked heterogeneity in MASLD severity and progression. Collectively, they link systemic insulin resistance to hepatic lipid accumulation and fibrogenesis, explaining the increased severity in impaired glucose tolerance and type 2 diabetes.

### Insulin secretion and hepatic clearance in MASLD

MASLD is deeply intertwined with systemic insulin dynamics. In early MASLD, compensatory hyperinsulinaemia reflects adaptive beta cell responses to peripheral insulin resistance, with increased basal insulin secretion and prolonged postprandial insulin output maintaining glucose homeostasis [56]. While traditionally interpreted as a compensatory response to insulin resistance, the temporal and causal relationship remains debated, as it is not currently possible to definitively determine whether hyperinsulinaemia precedes or follows the development of insulin resistance in humans (see [8]). Nonetheless, in early MASLD, beta cell function relative to insulin sensitivity, captured by the disposition index (which integrates insulin secretion with the degree of insulin resistance), remains largely preserved [57, 59], indicating that hyperinsulinaemia effectively compensates for mild to moderate insulin resistance, maintaining homeostasis despite underlying metabolic stress. As disease progresses, individuals who develop MASH exhibit a reduced disposition index, indicating impaired beta cell compensation relative to insulin resistance and signalling a transition from adaptive hyperinsulinaemia to beta cell dysfunction [57]. Thus, this apparent compensation masks underlying beta cell dysregulation: chronic metabolic stress, including lipotoxicity associated with hepatic fat accumulation, can blunt beta cell secretory responsiveness over time, reducing the secretory reserve and contributing to deteriorating glucose management and disease progression [57].

Insulin secretion cannot be dissociated from insulin clearance. The metabolic clearance rate of insulin declines in parallel with increased insulin secretion in insulin-resistant states, such that circulating insulin concentrations are a composite of secretion and clearance adaptations [60]. Insulin is secreted into the portal vein and approximately 70–80% of portal insulin is cleared on first pass through the liver (reviewed by Najjar et al [61]). Reduced clearance is consistently observed with increased liver fat content, independent of total adiposity [12, 58, 59, 61]. Hepatic clearance is mediated predominantly by receptor-mediated endocytosis involving carcinoembryonic antigen-related cell adhesion molecule 1 (CEACAM1) and proteolytic degradation by the insulin-degrading enzyme (IDE) [61]. Reduction in clearance mechanisms, particularly hepatic first-pass extraction, can play a role in increasing circulating insulin levels, which acts as a compensatory mechanism to maintain euglycaemia in the presence of insulin resistance [60, 61]. In humans, circulating (soluble) CEACAM1 levels are positively correlated with insulin clearance rates, suggesting that reduced CEACAM1 levels reflect impaired hepatic extraction of insulin and are a marker of poor metabolic health [62].

Reduced hepatic clearance in MASLD may also be exacerbated by microvascular changes and portosystemic shunting in advanced disease, whereby insulin bypasses the hepatic extraction sites, elevating peripheral insulin exposure [63]. However, genetic and causal inference studies indicate that hepatic steatosis itself is unlikely to be a primary causal determinant of impaired hepatic insulin clearance [64]; rather, other components of the metabolic syndrome, in particular insulin resistance, may cause peripheral hyperinsulinaemia by decreasing hepatic insulin extraction [65], suggesting that defects in insulin clearance more likely reflect systemic metabolic dysfunction and disease stage rather than directly driving steatosis.

### Glucagon secretion and action in MASLD

In MASLD, fasting hyperglucagonaemia coexists with hyperinsulinaemia and occurs independently of BMI, type 2 diabetes and degree of hepatic fibrosis [66]. Importantly, hyperglucagonaemia in MASLD is not simply a consequence of increased secretion, but reflects impaired hepatic glucagon action, that is, glucagon resistance.

Mechanistically, hepatic glucagon responsiveness is differentially preserved. Glucose-regulating actions remain intact; thus, glucagon continues to stimulate hepatic glucose output via glycogenolysis and gluconeogenesis [28], the principal determinants of fasting hyperglycaemia [26]. In contrast, glucagon regulation of amino acid metabolism and ureagenesis appears more complex and is potentially context dependent. However, direct functional studies indicate that glucagon-stimulated amino acid metabolism is largely preserved in individuals with hepatic steatosis [67]. In contrast, experimental models suggest that hepatic steatosis may impair glucagon-stimulated ureagenesis and disrupt the liver–alpha cell axis, contributing to altered amino acid–glucagon feedback signalling [68].

Taken together, these findings suggest that glucagon action is selectively dysregulated in MASLD. While glucose-regulatory effects are preserved, components of amino acid handling and ureagenesis may be variably affected. This functional divergence is likely to reflect an underlying insulin–glucagon imbalance that favours hepatic glucose overproduction and altered hepatic substrate partitioning. At the systemic level, this hormonal imbalance promotes a catabolic state characterised by increased proteolysis, enhanced lipid turnover and sustained metabolic stress. Over time, these adaptations may lead to progressive metabolic dysfunction and an increased risk of type 2 diabetes [69].

Hyperglucagonaemia in MASLD is further sustained by a feed-forward mechanism driven by the liver–pancreas axis. Impaired hepatic amino acid uptake and ureagenesis increase circulating glucagonotropic amino acids, which chronically stimulate pancreatic alpha-cells [68].

Beyond glucose and amino acid regulation, emerging clinical evidence also implicates impaired glucagon signalling in hepatic lipid accumulation. Reduced glucagon action limits hepatic lipid oxidation and ketogenesis, favouring triacylglycerols retention, particularly when VLDL export is insufficient [28]. Glucagon receptor antagonism has been consistently associated with reduced FAO, increased hepatic steatosis and elevated liver enzyme levels [70], and in humans a loss-of-function mutation in *GCGR*, the gene encoding the glucagon receptor, has been linked to early-onset hepatic steatosis and cirrhosis [71]. Together, these findings support a model in which intact hepatic glucagon signalling is required not only for glucose homeostasis but also for maintaining lipid and amino acid metabolic balance, with selective disruption potentially contributing to MASLD pathogenesis.

### Insulin–glucagon imbalance as a driver of MASLD progression

The disruption in insulin–glucagon balance in MASLD represents the unifying mechanism linking insulin resistance, altered glucagon action and hepatic lipid accumulation. Both hormones influence Kupffer cell activation, macrophage polarisation and HSC behaviour, thereby integrating metabolic and immune pathways that drive fibrogenesis [2, 37]. In this context, hormonal imbalance functions as a central integrative signal rather than a simple reflection of circulating hormone levels, coordinating hepatocellular stress responses with non-parenchymal cell activation, thereby amplifying fibrogenic cascades.

Chronic hyperinsulinaemia, driven by systemic insulin resistance, compensatory pancreatic beta cell hypersecretion and reduced hepatic insulin clearance, is a hallmark of MASLD and correlates with disease severity [57, 58, 72]. Mechanistically, hyperinsulinaemia promotes de novo lipogenesis (DNL) in both humans and rodents via the Akt/mTORC1–SREBP1c pathway [73–75], enhancing hepatic lipid accumulation and the synthesis of lipotoxic lipids (e.g. ceramides) that trigger stress responses and the release of profibrogenic mediators (e.g. TGF-β) [2, 29, 37, 45].

Beyond its metabolic role, hyperinsulinaemia has been shown to directly activate HSCs through phosphoinositide 3-kinase (PI3K)/Akt and MAPK pathways, and it stimulates type I collagen synthesis via α5β1 integrin-mediated signalling, independently of PI3K [76, 77]. Additional indirect effects are mediated through exacerbated oxidative stress and inflammation [63, 77, 78] (Fig. 2). While the clinical relevance of these direct profibrogenic effects remains a subject of debate, it is essential to distinguish the pathophysiological impact of endogenous hyperinsulinaemia from that of insulin therapy. For instance, the ORIGIN trial demonstrated that exogenous insulin therapy does not increase mitogenic risk or adverse cardiovascular outcomes in individuals with dysglycaemia [79]. Furthermore, while therapeutic insulin can improve hepatic insulin sensitivity and reduce steatosis [80], it is frequently associated with weight gain and increased visceral and subcutaneous adiposity [80, 81]. Consequently, chronic endogenous hyperinsulinaemia serves as a central driver of the metabolic and profibrogenic milieu that characterises MASLD progression, distinct from the effects observed with clinical insulin administration.

Impaired insulin clearance further contributes to disease progression. Hepatic CEACAM1 expression, a key mediator of insulin clearance, declines with MASH progression in humans, and hepatocyte-specific deletion of CEACAM1 is sufficient to induce hepatic fibrosis in experimental models, even in the absence of primary contributions from non-parenchymal cells [82]. Higher fasting insulin levels are associated with a lower likelihood of spontaneous fibrosis regression in the longitudinal NASH Clinical Research Network observational study [83], supporting a direct link between hyperinsulinaemia, impaired insulin clearance and fibrogenesis, as also proposed by others [63].

Concurrently, impaired glucagon action exacerbates hepatic metabolic flexibility by limiting lipid oxidation and amino acid disposal, thereby sustaining hepatocellular stress and reinforcing alpha cell hypersecretion. Together, insulin excess and altered glucagon action may converge to amplify metabolic stress, inflammation and fibrogenic signalling, positioning the insulin–glucagon axis as a central driver of MASLD progression and a compelling therapeutic target.

### Adipo-IR index as a marker of fibrosis

AT insulin resistance has emerged as a key correlate of hepatic metabolic dysfunction and disease severity in MASLD [10, 78, 84]. In this context, the AT insulin resistance index (Adipo-IR, calculated as fasting NEFA concentration × fasting insulin concentration) has been used as a surrogate marker of impaired adipose antilipolytic insulin action [55]. Notably, Adipo-IR has shown consistent associations with histological disease severity in biopsy-proven MASLD and MASH cohorts [10, 84–88]. Compared with indirect indices of adiposity or dyslipidaemia, such as the hypertriglyceridaemic waist (HTGW) index or the visceral adiposity index (VAI), Adipo-IR more directly reflects dysfunctional adipose lipid handling. Moreover, the relationships between the HTGW index and VAI and AT insulin resistance and liver fibrosis remain inconsistent or insufficiently validated [89, 90].

Although direct causality between AT insulin resistance and liver fibrosis in humans remains difficult to establish, converging lines of evidence support a contributory role of adipose dysfunction in driving hepatic injury. In individuals with MASLD, excess NEFA delivery, largely derived from AT lipolysis, promotes lipotoxic stress, oxidative injury and inflammatory signalling pathways that are implicated in hepatocellular damage and fibrogenesis, as reviewed in [91] (Figs 1, 2). The mechanism by which NEFAs induce hepatic insulin resistance has been examined in both animal and human studies. Lam et al [92] demonstrated in rats that elevation of plasma NEFAs via heparinised lipid infusion activates PKC-δ in the liver. In a complementary study, Boden et al [93] showed in conscious male rats using euglycaemic–hyperinsulinaemic clamps that high NEFA levels induce hepatic insulin resistance, associated with increased hepatic DAG content and activation of serine/threonine kinases, including PKC-δ and IKK-β.

In humans, Roden et al [94] demonstrated using lipid infusion and clamp techniques that elevation of plasma NEFAs induces hepatic insulin resistance. Similarly, Boden et al showed that increased lipid availability impairs hepatic glucose metabolism in healthy individuals [95]. These findings support a liver-specific lipid-mediated mechanism involving intracellular DAG accumulation and activation of novel PKC isoforms, which disrupts insulin signalling and leads to hepatic insulin resistance. Collectively, these data from rodent models and human metabolic studies support a unified mechanism in which elevated plasma NEFAs promote hepatic lipid accumulation, particularly DAG, leading to activation of PKC isoforms and downstream serine/threonine kinase signalling. This results in impaired insulin receptor signalling and reduced insulin-mediated suppression of hepatic glucose metabolism, establishing ectopic lipid-induced PKC activation as a central pathway in hepatic insulin resistance. These conclusions are further supported by human perfused liver studies showing that NEFAs directly impair hepatic insulin signalling and key phosphorylation steps in the insulin signalling cascade [96].

Dysfunctional AT also exhibits endocrine and inflammatory alterations, including reduced secretion of adiponectin and increased secretion of leptin and proinflammatory cytokines [18]. These changes are particularly pronounced in individuals with MASLD and contribute to worsening insulin resistance, hepatic inflammation [44, 97, 98] and activation of HSCs, driving hepatic fibrosis [37, 39, 99].

Interventional studies with pioglitazone have demonstrated that improvements in Adipo-IR correlate with reductions in steatosis, inflammation and fibrosis, supporting a relationship between improved adipose insulin sensitivity and attenuation of liver injury [85, 86]. In a large contemporary cohort with type 2 diabetes and MASLD, Adipo-IR was independently associated with clinically significant hepatic fibrosis assessed by elastography, outperforming conventional measures of hepatic insulin resistance and overall adiposity [84].

### Steroids and sex hormones as modulators of inflammation and fibrosis

Steroid hormones, including cortisol, oestrogen and testosterone, play key roles in regulating hepatic and systemic metabolism, and their dysregulation contributes to MASLD pathogenesis.

Cortisol, the principal glucocorticoid, promotes hepatic gluconeogenesis, stimulates DNL, exacerbates insulin resistance and impairs AT function. Beyond these metabolic effects, preclinical studies indicate that cortisol directly enhances hepatic inflammation and fibrogenesis, accelerating disease progression beyond simple steatosis [100]. Tissue-specific regulation of glucocorticoid availability is critical: 11beta-hydroxysteroid dehydrogenase type 1 (11β-HSD1) regenerates active cortisol locally in liver and AT, amplifying glucocorticoid action, whereas 5α-reductase converts cortisol into inactive metabolites, limiting hepatic exposure. Dysregulation of these enzymes has been associated with MASLD severity, and experimental inhibition of 11β-HSD1 or modulation of hepatic cortisol metabolism attenuates steatosis, inflammation and fibrosis, supporting a pathogenic role for excessive intrahepatic glucocorticoid activity [101, 102].

In addition, sex-specific adipose biology shapes adipose–liver crosstalk, insulin resistance and MASLD susceptibility [103]. At comparable BMI, men and postmenopausal women display increased VAT accumulation and hepatic triacylglycerols storage and early insulin resistance, whereas premenopausal women preferentially expand SAT while maintaining insulin sensitivity, thereby buffering circulating NEFAs and limiting hepatic lipid spillover [103]. Lipidomic analyses have identified sex-specific circulating lipid signatures, reflecting qualitative differences in lipid handling and hepatic metabolic adaptability [104]. Higher adiponectin levels in women further support insulin sensitivity and may protect against hepatic inflammation and fibrosis [103, 105].

Within this sex-specific metabolic framework, oestrogen and testosterone exert distinct and context-dependent effects on AT distribution, hepatic metabolism and fibrogenic pathways. Oestrogen exerts protective effects on liver and metabolic health in animal models by enhancing lipid storage in SAT, supporting mitochondrial activity and FAO, maintaining insulin sensitivity and suppressing hepatic DNL [106–108]. The menopausal transition represents a critical shift in this axis. In postmenopausal women, declining oestrogen levels are associated with redistribution of fat from SAT towards VAT, impaired suppression of lipolysis, lower adiponectin concentrations and a more proinflammatory adipose milieu [109, 110]. These changes increase systemic and portal NEFA flux, promote hepatic triacylglycerols accumulation and may weaken or abolish the relative protection against MASLD observed in premenopausal women [110].

The potential protective role of oestrogen is further supported by a recent large-scale retrospective cohort study involving perimenopausal women with pre-existing MASLD [111]. Using propensity score matching to compare hormone replacement therapy (HRT) users against untreated control participants, the study found that HRT was associated with a significantly lower risk of major adverse liver outcomes (MALO), including portal hypertension, cirrhosis and hepatocellular carcinoma. However, as retrospective data are subject to inherent selection biases, prospective validation is essential. To this end, several clinical trials are currently underway. The COMHO-MASLD trial (NCT06704516) is a prospective mechanistic study specifically investigating how the initiation of HRT in postmenopausal women affects hepatic lipid content and metabolic drivers of fat accumulation. Another trial (NCT04833140) is evaluating the impact of direct oestradiol administration on liver fat and fibrosis markers in postmenopausal women with confirmed MASH. Until such randomised placebo-controlled trials are completed, the use of oestrogen for liver-specific protection remains an area of active investigation rather than standard clinical practice.

Testosterone influences MASLD risk in a sex-dependent manner. Androgen excess in women (e.g. in polycystic ovary syndrome, now termed polyendocrine metabolic ovarian syndrome) and age-related testosterone deficiency in men are associated with adverse AT remodelling, VAT expansion, insulin resistance and hepatic lipid accumulation [112, 113].

Together, steroid and sex hormones interact with depot-specific adipose biology to modulate the metabolic milieu and intrahepatic glucocorticoid activity. These mechanisms contribute to MASLD heterogeneity and underlie sex-specific differences in susceptibility, progression and metabolic response.

## Metabolic hormones as therapeutic targets in MASLD

### Thyroid hormone signalling as a regulator of hepatic lipotoxicity and fibrosis

Thyroid hormones are key regulators of basal metabolic rate, lipid metabolism and cholesterol homeostasis, acting predominantly through the liver-enriched thyroid hormone receptor-β (THR-β) isoform. Individuals with low thyroid function, including subclinical hypothyroidism, have a higher prevalence of MASH, and the risks of MASH and advanced fibrosis increased significantly with increasing plasma thyroid-stimulating hormone (TSH) concentrations [114]. Impaired hepatic thyroid hormone signalling has also been demonstrated in liver tissues of individuals with MASLD, mirroring the preclinical data showing defective lipid use, increased DNL and intrahepatic lipid accumulation [115].

**Fig. 3:**
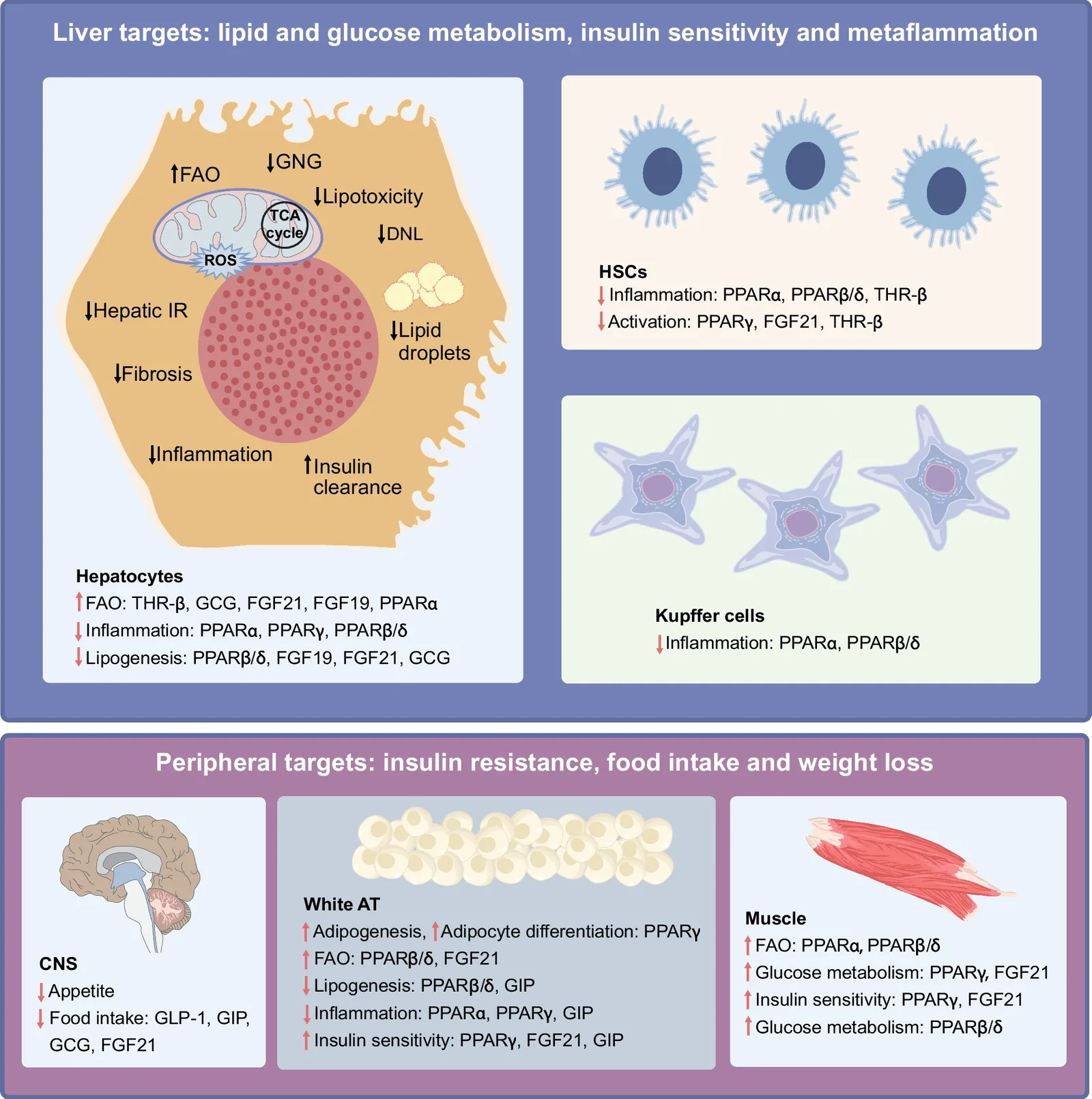
Main direct and indirect targets for therapeutic modulation of liver dysfunction in MASLD/MASH. Improvements in dysmetabolic processes underlying MASLD and MASH, including AT dysfunction, liver steatosis, defective FAO, lipotoxicity, insulin resistance (IR), inflammation and fibrosis, can be achieved through direct modulation of hepatic signalling pathways or indirect modulation of central nervous system, adipose and muscle signalling pathways involving nuclear receptors such as peroxisome proliferator-activated receptors (PPARα, β/δ and γ), incretins (glucagon-like peptide-1 [GLP-1] and glucose-dependent insulinotropic polypeptide [GIP]), fibroblast growth factors (FGF-19 and FGF-21) and THR-β. CNS, central nervous system; GCG, glucagon; GNG, gluconeogenesis; ROS, reactive oxygen species. This figure is available as part of a downloadable slideset

Mechanistically, THR-β activation enhances mitochondrial FAO, suppresses hepatic lipogenic pathways by downregulating SREBP1c-mediated DNL and enhancing mitochondrial lipid oxidation, and promotes cholesterol clearance, while minimising extrahepatic thyroid-related adverse effects, as reviewed in [116]. Moreover, THR-β activation enhances bile acid synthesis and LDL receptor expression, resulting in significant reductions in circulating LDL-cholesterol and overall cardiometabolic risk. Beyond lipid lowering, THR-β agonists exert indirect antifibrotic effects by reducing hepatocellular lipotoxicity, inflammation and oxidative stress, thereby limiting HSC activation and extracellular matrix deposition. The positive results of the MAESTRO trial [117] have led to the regulatory approval of resmetirom, a THR-β agonist, as the first targeted antifibrotic therapy for MASH. By integrating antifibrotic effects with improvements in steatosis and cardiometabolic risk, THR-β agonists represent a mechanism-based disease-modifying therapy for MASH (Fig. 3) (see [118]).

### Incretin hormones as indirect modulators of hepatic inflammation and fibrosis

Glucagon-like peptide-1 (GLP-1) and glucose-dependent insulinotropic polypeptide (GIP) are gut-derived incretin hormones secreted in response to nutrient intake that enhance glucose-dependent insulin secretion and regulate appetite and energy balance [119] (Fig. 3). In metabolic disorders, including MASLD and MASH, impaired incretin signalling contributes to hyperglycaemia, insulin resistance, dysregulated lipid metabolism and hepatic lipid overload [120].

GLP-1 receptor agonists (GLP-1RAs) restore incretin function through both central and peripheral mechanisms: they reduce appetite and energy intake, leading to sustained weight loss, reduce hyperglycaemia and hepatic steatosis, and induce resolution of MASH [119]. The GLP-1RA semaglutide has been approved in the USA and Europe for the treatment of non-cirrhotic MASH with moderate or advanced liver fibrosis (stage F2 or F3) (see [118]).

The mechanistic effects of GLP-1RAs in the liver remain uncertain owing to the lack of GLP-1 receptor expression on human hepatocytes, Kupffer cells or HSCs [119]; however, treatment of individuals with MASH with liraglutide reduced HGP, DNL and hepatic lipid accumulation, and stimulated lipolysis [88]. The effect of GLP-1RAs on HGP has been confirmed in other studies (reviewed in [121]), whereas no effect on lipolysis was observed after infusion of GLP-1 during fasting in healthy individuals [122] or after treatment with a GLP-1RA in the postprandial state in individuals with obesity [123], thus questioning a direct effect.

GLP-1RAs are also considered to have mainly indirect effects on fibrosis, that is, mediated by weight loss [124], which contributes by reducing lipotoxicity and attenuating hepatocellular stress and inflammatory signalling, thereby limiting downstream activation of HSCs and fibrogenic pathways.

However, more recent experimental evidence suggests the presence of additional intrahepatic, cell-specific mechanisms. In a murine MASH model, semaglutide improved steatosis, fibrosis and immune remodelling even without weight loss. GLP-1 receptor expression was identified in liver sinusoidal endothelial cells (LSECs) and CD8^+^ T cells. Endothelial GLP-1 receptor signalling was required for full hepatic benefits; its deletion or knockdown reduced efficacy despite preserved weight loss. LSECs also showed a stress-responsive phenotype in MASH that was reversed by semaglutide. These data support a weight loss-independent, endothelial cell-mediated intrahepatic mechanism of GLP-1RA action [125].

Evidence also suggests that targeting multiple incretin pathways (GLP-1/GIP or GLP-1/glucagon) increases the number of responders and achieves greater reductions in hepatic steatosis [126]. With regard to GIP, the improvement in hepatic metabolism is likely to be indirect, as GIP receptors are not present in the liver but are abundant in AT, where GIP improves metabolism and lipid storage [119], whereas glucagon has direct effects on the liver as discussed in ‘Glucagon secretion and action in MASLD’.

### PPAR activation as a therapeutic modulator of metabolism and fibrogenesis

Peroxisome proliferator-activated receptor (PPAR) agonists improve insulin sensitivity and modulate lipid and inflammatory pathways (Fig. 3). Initially developed for type 2 diabetes owing to their insulin-sensitising effects, they are now recognised as effective therapies for MASLD and MASH [127].

PPARs are widely expressed across metabolic tissues, including hepatocytes, Kupffer cells and HSCs, where activation of different isoforms regulates complementary pathways in lipid handling and inflammation (reviewed in [128]). Hepatic PPARα activation promotes FAO, mitochondrial activity, ketogenesis and lipoprotein metabolism, thereby reducing steatosis and inflammation; in contrast, PPARγ primarily regulates lipid storage and exerts anti-inflammatory effects, and PPARβ/δ contributes to the suppression of DNL and inflammatory signalling [128].

In individuals with MASLD, hepatic expression of PPARα is often reduced; however, targeting only PPARα (e.g. with fibrates) has no effect on reducing hepatic steatosis or improving histology in individuals with MASLD/MASH [128]. In humans, PPARγ agonists, such as the thiazolidinedione pioglitazone, enhance AT insulin sensitivity, reduce NEFA flux to the liver and improve adipokine signalling, collectively decreasing hepatic steatosis and inflammation [85, 86, 129, 130]. However, thiazolidinediones, while effective, are limited by side effects of weight gain, fluid retention and increased fracture risk, although low-dose regimens may mitigate these effects while preserving hepatic benefits [131]. In addition to metabolic effects, activation of multiple PPAR isoforms can exert anti-fibrogenic actions by modulating HSC activation and shaping Kupffer cell and macrophage phenotypes towards less proinflammatory and profibrotic states, thereby contributing to attenuation of liver fibrosis [132–135] (Fig. 3).

Dual PPAR agonists, such as saroglitazar (a PPARα/γ agonist), and pan-PPAR agonists (PPARα/γ/δ), such as lanifibranor, target multiple PPAR isoforms, promoting broader metabolic reprogramming. Saroglitazar has recently been approved in India for MASH and is currently being evaluated in a global Phase IIb trial (NCT05011305). Lanifibranor is in Phase III development (NATiV3 trial, NCT04849728) and has shown promising reductions in liver fibrosis in the Phase IIb NATIVE trial [128], representing a potential disease-modifying strategy that simultaneously addresses metabolic dysfunction, steatohepatitis, inflammation and fibrogenesis in MASLD and MASH.

### Fibroblast growth factors as endocrine regulators of fibrosis and metabolic homeostasis

Fibroblast growth factors, notably FGF21 and FGF19, are endocrine hormones that coordinate systemic energy and lipid metabolism (reviewed in [136]). FGF21 is primarily secreted by the liver in response to metabolic stress and acts on multiple targets. Peripherally, it signals through FGFR1c–β-Klotho complexes in AT to enhance insulin sensitivity, promote FAO and reduce lipotoxicity, indirectly benefiting hepatic metabolism. Additionally, FGF21 acts on the central nervous system, particularly the hypothalamus, to suppress appetite, increase energy expenditure and reduce body weight, further alleviating hepatic steatosis (Fig. 3). Preclinical studies and early-phase clinical trials indicate that FGF21 agonism also attenuates hepatic inflammation and fibrogenesis, partly by reducing hepatocellular stress and limiting activation of HSCs [136].

FGF19 is secreted by ileal enterocytes in response to postprandial bile acid signalling via the FXR, illustrating the concept of bile acids as metabolic hormones. FXR activation in the intestine induces the expression of FGF19 (the human orthologue of rodent FGF15), which enters the circulation and activates the receptor complex FGFR4–β-Klotho on hepatocytes. This pathway suppresses CYP7A1-mediated bile acid synthesis, reduces hepatic DNL, modulates cholesterol metabolism and improves liver lipid handling. Dysregulation of FXR–FGF19 signalling in MASLD/MASH contributes to impaired lipid disposal, reduced metabolic flexibility and disease progression [137].

Therapeutic FGF analogues, including pegbelfermin and pegozafermin (FGF21), efruxifermin (FGF21 fusion protein) and aldafermin (FGF19) restore these pathways [136], consistently reducing hepatic steatosis, improving insulin sensitivity, promoting weight loss and favourably modulating inflammatory and fibrogenic signalling. By integrating hepatic, adipose and central nervous system effects, and leveraging the FXR–FGF19 bile acid axis, these agents exemplify endocrine-directed therapies that address the multisystem metabolic drivers of MASLD and MASH (Fig. 3).

## Conclusion

MASLD and MASH arise from systemic metabolic dysfunction in which AT dysfunction and hormonal imbalance converge to drive hepatic steatosis, inflammation and fibrosis. Interorgan crosstalk amplifies disease progression. Targeting these upstream metabolic and hormonal pathways, through agents that restore the insulin–glucagon balance, improve lipid metabolism and/or modulate fibrogenic signalling, offers a precision medicine approach to prevent progression, resolve MASH and limit fibrosis.

## Supplementary Information

Below is the link to the electronic supplementary material.

Slideset of figures (PPTX 871 KB)

## Abbreviations

AT: Adipose tissue
CEACAM1: Carcinoembryonic antigen-related cell adhesion molecule 1
DAG: Diacylglycerol
DNL: De novo lipogenesis
FAO: Fatty acid oxidation
FGF: Fibroblast growth factor
FXR: Farnesoid X receptor
GIP: Glucose-dependent insulinotropic polypeptide
GLP-1: Glucagon-like peptide-1
GLP-1RA: Glucagon-like peptide-1 receptor agonist
HGP: Hepatic glucose production
HSC: Hepatic stellate cell
MASLD: Metabolic dysfunction-associated steatotic liver disease
MASH: Metabolic dysfunction-associated steatohepatitis
PKC: Protein kinase C
PPAR: Peroxisome proliferator-activated receptor
SAT: Subcutaneous adipose tissue
TCA: Tricarboxylic acid
THR-β: Thyroid hormone receptor-β
TLR: Toll-like receptor
VAT: Visceral adipose tissue
